# Engineering *Pseudomonas putida* KT2440 for simultaneous degradation of carbofuran and chlorpyrifos

**DOI:** 10.1111/1751-7915.12381

**Published:** 2016-07-15

**Authors:** Ting Gong, Ruihua Liu, You Che, Xiaoqing Xu, Fengjie Zhao, Huilei Yu, Cunjiang Song, Yanping Liu, Chao Yang

**Affiliations:** ^1^ Key Laboratory of Molecular Microbiology and Technology for Ministry of Education and State Key Laboratory of Medicinal Chemical BiologyNankai UniversityTianjin300071China; ^2^ State Key Laboratory of Bioreactor EngineeringEast China University of Science and TechnologyShanghai200237China; ^3^ Department of Gynaecology and ObstetricsTianjin Medical University General HospitalTianjin300052China

## Abstract

Currently, chlorpyrifos (CP) and carbofuran are often applied together to control major agricultural pests in many developing countries, in most cases, they are simultaneously detected in agricultural soils. Some cost‐effective techniques are required for the remediation of combined pollution caused by multiple pesticides. In this work, we aim at constructing a detectable recombinant microorganism with the capacity to simultaneously degrade CP and carbofuran. To achieve this purpose, CP/carbofuran hydrolase genes and *gfp* were integrated into the chromosome of a biosafety strain *Pseudomonas putida* KT2440 using a chromosomal scarless modification strategy with *upp* as a counter‐selectable marker. The toxicity of the hydrolysis products was significantly lower compared with the parent compounds. The recombinant strain could utilize CP or carbofuran as the sole source of carbon for growth. The inoculation of the recombinant strain to soils treated with carbofuran and CP resulted in a higher degradation rate than in noninoculated soils. Introduced green fluorescent protein can be employed as a biomarker to track the recombinant strain during bioremediation. Therefore, the recombinant strain has potential to be applied for *in situ* bioremediation of soil co‐contaminated with carbofuran and CP.

## Introduction

Chlorpyrifos (CP) is a moderately toxic organophosphate insecticide that exhibits broad‐spectrum activity against major agricultural pests. CP leads to the loss of nerve function by irreversible inhibition of acetylcholinesterase (AChE) (Singh, 2009). Carbofuran is a broad‐spectrum carbamate insecticide that is used widely for disease control in vegetable, fruit and crops. However, the potential health risks to humans posed by excessive use of carbofuran are not ignored because carbofuran is a potent AChE inhibitor (Nguyen *et al*., 2014). Carbofuran and CP are often applied to agricultural soil together, thus, combined pollution caused by them needs to be resolved by developing new techniques (Rama Krishna and Philip, 2011).

Microbial degradation plays important roles in the removal of various pesticides in soil. Several carbofuran‐ and CP‐degrading bacteria have been isolated from natural environments (Tomasek and Karns, 1989; Feng *et al*., 1997; Ogramab *et al*., 2000; Yang *et al*., 2006; Li *et al*., 2007; Xu *et al*., 2008; Anwar *et al*., 2009). Moreover, carbofuran hydrolase gene (*mcd* encoding CH) and CP hydrolase gene (*mpd* encoding MPH) have been cloned from *Achromobacter* sp. WM111 and *Stenotrophomonas* sp. YC‐1 respectively (Tomasek and Karns, 1989; Yang *et al*., 2006). Hydrolysis of carbofuran and CP by the hydrolases reduces their toxicity by several orders of magnitude (Hernándeza *et al*., 2013), providing a useful enzymatic detoxification method. To date, there are no reports on simultaneous degradation of carbofuran and CP by a single microorganism.

Generally, it takes more effort to simultaneously cultivate multiple pesticide‐degrading bacteria with different culture conditions. When the degraders are applied for *in situ* bioremediation, the different niches of multiple independent degraders force them to compete limited resources, resulting in the unbalanced growth of the degraders. More importantly, the biosafety of various natural degraders is uncertain, which will give rise to potential risks to environmental safety and human health. An alternative solution is to create a recombinant strain capable of simultaneously degrading carbofuran and CP. Generally, the recombinant strains harbouring degradation genes have higher degradation rates than natural degraders by introducing minimal gene expression regulation elements to promote high‐level expression of degradation genes.

So far, several recombinant strains have been constructed for degradation of different classes of pesticides. Among them, a recombinant strain capable of simultaneously degrading methyl parathion and carbofuran was constructed by random insertion of a methyl parathion hydrolase (*mph*) gene into the chromosome of a carbofuran‐degrading *Sphingomonas* sp. CDS‐1 using a mini‐Tn5 transposon system. Unfortunately, antibiotic resistance gene (ARG) left on chromosome has potential risks to environmental safety due to ARG diffusion among bacterial species by horizontal gene transfer (Jiang *et al*., 2007). In addition, a γ‐hexachlorocyclohexane (γ‐HCH)‐degrading *Sphingobium japonicum* UT26 was endowed with the capacity to simultaneously degrade γ‐HCH and organophosphates by transforming with a plasmid encoding an organophosphorus hydrolase. However, plasmid instability reduces the efficacy of the recombinant stain for bioremediation of contaminated soil (Yang *et al*., 2013).

A model microorganism *Pseudomonas putida* KT2440 was certified by the Recombinant DNA Advisory Committee as a biosafety strain for recombinant DNA manipulation (Nelson *et al*., 2002). *P. putida* KT2440 is a robust soil bacterium and is capable of degrading diverse aromatic compounds (Jiménez *et al*., 2002). The genome of *P. putida* KT2440 has been sequenced and the chromosomal scarless modification methods have been well established in *P. putida* KT2440 (Nelson *et al*., 2002; Graf and Altenbuchner, 2011; Luo *et al*., 2016), which facilitate the genetic engineering of *P. putida* KT2440 to acquire novel characteristics for biodegradation and biocatalysis. Recently, *P. putida* KT2440 has been highlighted as an optimal chassis for metabolic pathway assembly (Nikel *et al*., 2014).

In this study, *P. putida* KT2440 was endowed with carbofuran/CP degradation capability and a biomarker by integrating *mcd*,* mpd* and *gfp* into the chromosome using a genomic scarless modification method. This approach allows successive insertion of various degradation genes into the chromosome of *P. putida* KT2440 to create extended‐spectrum degraders. Moreover, the recombinant strain marked with green fluorescent protein (GFP) may be monitored by fluorescence during bioremediation.

## Results and discussion

### Construction of recombinant *P. putida* KT2440 for simultaneous degradation of carbofuran and CP

Currently, combined pollution caused by multiple pesticides has become an urgent issue to be resolved. To date, two approaches to remediate multiple pesticides‐contaminated soils have been proposed, including the introduction of different degradation genes into a host strain and the use of multiple natural degraders with different substrate ranges (Singh and Walker, 2006). In this work, a biosafety strain *P. putida* KT2440 was selected as a host for heterologous expression of carbofuran and CP hydrolase genes. For this purpose, a genome markerless modification method based on the *upp* counter‐selection system (Gong *et al*., 2016) was used to construct the recombinant strain with the capacity to simultaneously degrade carbofuran and CP. The synthetic gene cassette (*mpd*,* gfp* or *mcd*) was composed of a *P. putida* constitutive promoter J23119, a ribosomal binding site and an exogenous gene flanked by the upstream and downstream homologous arms (Fig. S1). Successive insertion of the *mpd*,* gfp* and *mcd* genes into three different target sites on the chromosome of *P. putida* KT2440 was achieved with the suicide plasmid in combination with the *upp* counter‐selection system. The successful constructions were verified by PCR detection of the inserted gene cassettes (Fig. S2). The resulting recombination strain was designated as *P. putida* KTU‐PGC.

### Transcription of exogenous genes in *P. putida* KTU‐PGC

A *P. putida* constitutive promoter J23119 has been demonstrated to be efficient for the constitutive expression of exogenous genes in *P. putida* (Shetty *et al*., 2008). In this study, expression of *mpd*,* gfp* and *mcd* genes was under the control of the constitutive promoter J23119. The transcription of the inserted exogenous genes (*mpd*,* gfp* and *mcd*) in *P. putida* KTU‐PGC was further analysed by RT‐PCR assays with the extracted mRNA as the template. As a result, the desired target fragments were obtained by PCR with the cDNA or genomic DNA as the template. In contrast, no positive results were observed with the mRNA or ddH_2_O as the template (Fig. S3). These results indicated that the introduced exogenous genes were successfully transcribed to mRNA in *P. putida* KTU‐PGC.

### Simultaneous degradation of carbofuran and CP by *P. putida* KTU‐PGC

**Figure 1:**
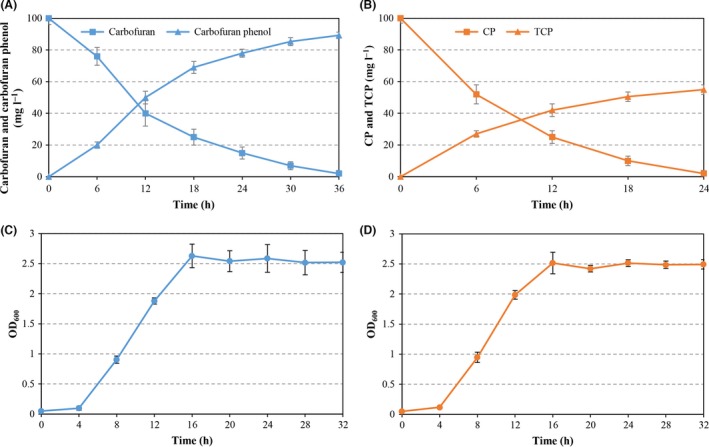
(A and B) Simultaneous degradation of carbofuran and CP by *Pseudomonas putida* KTU‐PGC. Degradation experiments with *P. putida* KTU‐PGC were performed at an initial inoculum density of OD_600_ = 0.05 in M9 minimal medium supplemented with 100 mg l^−1^ carbofuran and CP at 30°C. (C and D) Cell growth of *P. putida* KTU‐PGC at 30°C in M9 minimal medium supplemented with 100 mg l^−1^ carbofuran or CP as the sole source of carbon.

To demonstrate the capacity of the recombinant strain to simultaneously degrade carbofuran and CP, the degradation experiments were performed by inoculating the recombinant strain into M9 minimal medium supplemented with 100 mg l^−1^ carbofuran and CP. High‐performance liquid chromatography (HPLC) analysis indicated that 100 mg l^−1^ carbofuran and CP were completely degraded within 36 and 24 h respectively (Fig. 1). Moreover, the concentration of carbonfuran phenol and 3,5,6‐trichloro‐2‐pyridinol (TCP) in M9 minimal medium increased gradually with the decrease in carbofuran and CP concentration. Both carbofuran and CP were degraded quickly at the first 12 h, which accounted for 60% and 75% of the amount of the initially added pesticides respectively. The reduction in the degradation rate after 12 h may be due to the accumulation of carbonfuran phenol and TCP, which have antimicrobial activity and are toxic to the bacterial growth and metabolism (Singh *et al*., 2004; Nguyen *et al*., 2014). In contrast, no decrease in the concentration of carbofuran and CP was detected in M9 minimal medium inoculated with *P. putida* KTU (data not shown).

When the recombinant strain was inoculated at OD_600_ = 0.05 into M9 minimal medium supplemented with 100 mg l^−1^ carbofuran or CP, the strain could utilize carbofuran or CP as the sole source of carbon for growth (Fig. 1).

In HPLC analysis of the cultures grown on 100 mg l^−1^ carbofuran, two peaks with a retension time (RT) of 8.75 and 10.21 min appeared in HPLC separation (Fig. S4), which were further identified as carbofuran and carbonfuran phenol by comparing with their authentic standards. HPLC analysis showed that carbofuran could be degraded by *P. putida* KTU‐PGC to carbonfuran phenol and methylamine (Fig. S5).

In HPLC analysis of the cultures grown on 100 mg l^−1^ CP, two peaks appeared at a RT of 8.69 and 3.12 min in HPLC separation (Fig. S6), which were further identified as CP and TCP by comparing with their authentic standards. HPLC analysis showed that CP could be degraded by *P. putida* KTU‐PGC to TCP and diethylthiophosphoric acid (Fig. S5).

### Functional expression of GFP in *P. putida* KTU‐PGC

**Figure 2:**
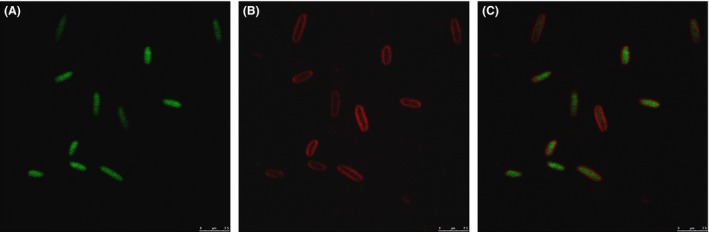
Detection of GFP fluorescence derived from *Pseudomonas putida* KTU‐PGC using a confocal microscope. A. Green fluorescence within the cell. B. Outline of cell membrane by stain with FM4‐64/L. C. A and B merged together.

To easily track the recombinant strain in the field‐scale remediation, a *gfp* gene was also inserted into the chromosome of *P. putida*, allowing direct fluorescent detection of the activity of the recombinant strain. Cells of the recombinant strain containing a chromosome‐borne GFP produced bright green fluorescence under a confocal microscope (Fig. 2), indicating the presence of active GFP in this strain. However, no fluorescence was observed with the control wild‐type strain. Therefore, GFP may be used as a biomarker to monitor the survival and movement of the recombinant strain during bioremediation.

### Genetic stability of engineered strain

*Pseudomonas putida* KTU‐PGC was successively subcultured for twenty generations at 30°C in LB medium. These subcultures degraded carbofuran and CP as fast as the parent strain. Results from PCR detection showed that the inserted exogenous genes (*mpd*,* gfp* and *mcd*) stably existed on the chromosome of the twentieth‐generation subcultures of *P. putida* KTU‐PGC (Fig. S7), which indicated that the engineered strain was genetically stable in the lack of selection pressure.

Nonessential genes of *P. putida* KT2440 were selected as the insertion sites (Table 1). To check whether the insertion of exogenous genes into the chromosome inhibits cell growth, the cell growth kinetics of *P. putida* KTU and KTU‐PGC were compared. No growth inhibition was observed for strain KTU‐PGC, which reached the final cell density similar to strain KTU after 48 h of incubation (Fig. S8), indicating that the inactivation of the nonessential genes had no negative influences on cell growth and metabolism.

**Table 1:** Information on three exogenous genes and their chromosomal insertion sites

Gene	Length (bp)	Amino acid residues	Function	Source (GenBank accession no.)	Insertion site
*mpd*	894	298	Chlorpyrifos hydrolase	*Stenotrophomonas* sp. YC‐1 (DQ677027.1)	PP_5003 (*phaA*)
*mcd*	1983	661	Carbofuran hydrolase	*Achromobacter* sp. WM111 (AF160188.1)	PP_1277/PP_1278 (*algA/algF*)
*gfp*	720	240	Green fluorescent protein	Plasmid pEGFP‐N3 (U57609)	PP_3357 (*vdh*)

### Remediation of soil co‐contaminated with carbofuran and CP by *P. putida* KTU‐PGC

**Figure 3:**
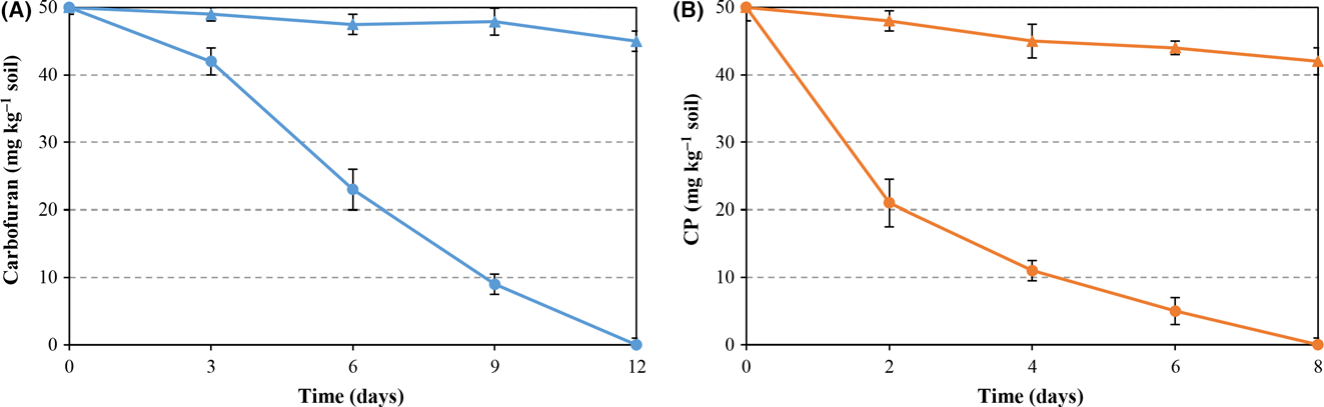
Simultaneous degradation of carbofuran and CP in soils inoculated with *Pseudomonas putida* KTU‐PGC at the rate of 10^6^ cells/g soil. Symbols: (●) soil, inoculated; (▲) soil, uninoculated.

To examine whether *P. putida* KTU‐PGC can thrive and effectively degrade carbofuran and CP in soil in the presence of indigenous microbial populations, soils co‐contaminated with carbofuran and CP were inoculated with *P. putida* KTU‐PGC at the rate of 10^6^ cells g^−1^ soil. During the 12‐day period, a slight reduction in carbofuran and CP was observed in soils without inoculation, most likely due to the occurrence of abiotic degradation processes. In contrast, carbofuran and CP were completely degraded in soils with inoculation within 12 and 8 days respectively (Fig. 3).

At the end of soil remediation experiments, soil samples were used for the isolation of inoculated *P. putida*. Two single colonies that produced clear zones on LB agar plates containing 50 mg l^−1^ CP were isolated and purified. The two isolates (named SKT‐A and SKT‐B) were identified as *P. putida* KT2440 by sequencing their 16S rRNA gene. Moreover, *mpd*,* gfp* and *mcd* genes were detected by PCR from the two isolates (Fig. S9). These results indicated that inoculated *P. putida* truly contributed to the degradation of carbofuran and CP in soil.

During the soil bioremediation experiments, we used GFP as a biomarker to track the presence and activity of the inoculated recombinant *P. putida* KT2440. The recombinant *P. putida* KT2440 was initially screened by PCR detection of the introduced *gfp* gene. Subsequently, the *gfp*‐containing strains were further measured for their fluorescence. As expected, the bacterial cells carrying *gfp* gene emitted green fluorescence under a confocal microscope (Fig. S10). Moreover, the recombinant *P. putida* KT2440 inoculated in soil could be monitored for its activity by measuring the changes in the fluorescence intensity during bioremediation. These results suggest that GFP may be a powerful real‐time monitoring tool for the visualization of the recombinant *P. putida* KT2440 during bioremediation.

### Potential of *P. putida* KT2440 for bioremediation of contaminated soil

Previously, a carbofuran‐degrading *Sphingomonas* sp. CDS‐1 was engineered to simultaneously degrade methyl parathion and carbofuran by randomly inserting a *mph* gene into the chromosome using a Tn5‐based transposon system (Jiang *et al*., 2007). However, random insertion on chromosome might disrupt essential genes in host strains, leading to function loss and cell death. In this study, exogenous genes were inserted into desirable regions on the chromosome of *P. putida* KT2440 by homologous recombination. Obviously, targeted insertion is more advantageous than random insertion. We selected nonessential genes of *P. putida* KT2440 as the insertion sites; thus, the insertion of exogenous genes neither inhibits cell growth nor affects cell survival. Compared with model strain *P. putida* KT2440, the biosafety of *Sphingomonas* sp. CDS‐1 needs to be intensively investigated prior to applying to soil remediation.

*P. putida* KT2440 possesses diverse catabolic pathways for aromatic compounds (Jiménez *et al*., 2002). Bacterial aromatic ring‐hydroxylating oxygenase database (RHObase) provides in‐depth information on functionally characterized RHOs from phylogenetically diverse classes of bacteria (Chakraborty *et al*., 2014), and therefore, RHObase is a useful tool to predict functionality of putative oxygenase from *de novo* genome or metagenome sequences. Distinctive bacterial communities associated with ectomycorrhizal roots were identified using 454 pyrosequencing (Marupakula *et al*., 2016). Recently, a rhizoremediation system based on an artificial consortium consisting of plant growth‐promoting rhizobacteria (PGPR) and polycyclic aromatic hydrocarbon‐degrading bacteria was used to remove petroleum wastes (Pizarro‐Tobías *et al*., 2015). *P. putida* KT2440 has been identified as PGPR and has an ability to colonize the rhizosphere of crop plants (Espinosa‐Urgel *et al*., 2002), which may facilitate the development of rhizoremediation systems for soil decontamination.

Synthetic biology has been highlighted as a powerful tool to create novel strains with desired degradative capabilities for bioremediation of contaminated environments (Gong *et al*., 2016). The recombinant *P. putida* KT2440 constructed in this study could efficiently degrade carbofuran and CP in soil and utilize carbofuran or CP as the sole source of carbon for growth.

Selection of *P. putida* KT2440 as host strain has many advantages, including confirmed biosafety, available whole‐genome sequence, and diverse genetic manipulation tools, which should lay a foundation for targeted chromosomal modification and metabolic pathway engineering in *P. putida* KT2440. *P. putida* KT2440 has been highlighted as an optimal chassis for implantation of various organic‐degrading genes to create a multifunctional engineered strain for simultaneous degradation of multiple contaminants (Gong *et al*., 2016). Recently, a two‐step markerless genome modification method was developed for *P. putida* KT2440, which combined λRed‐mediated homologous recombination with *Cre*‐recombinase catalysed site‐specific recombination (Luo *et al*., 2016). Graf and Altenbuchner (2011) developed a chromosomal marker‐free modification method for *P. putida* KT2440 by combining homologous recombination with *upp* counter‐selection system. In this work, CP/carbofuran hydrolase genes were inserted scarlessly into target sites on the chromosome of *P. putida* KT2440 using *upp* as a counter‐selectable marker. Release of genetically modified organisms (GMO) into the environment is strictly restricted due to serious concerns about the biosafety of GMO. *P. putida* KT2440 has strong competitiveness in soil microbial community (Espinosa‐Urgel *et al*., 2002). Most importantly, *P. putida* KT2440 has been shown to be a biosafety strain for *in situ* bioremediation (Nelson *et al*., 2002). The feasibility of applying the recombinant *P. putida* KT2440 for *in situ* bioremediation of soil co‐contaminated with carbofuran and CP is currently under investigation.

## Experimental procedures

### Reagents, strains and culture conditions

Carbofuran, CP, carbonfuran phenol and TCP (99% pure analytical grade) were purchased from Alta Scientific, Tianjin, China. Pesticides were stocked in methanol with a concentration of 10 mg ml^−1^. All the other chemical reagents were of analytical grade and purchased from Dingguo Biotechnology, Tianjin, China.

*Pseudomonas putida* strains were grown at 30°C in M9 minimal medium (Graf and Altenbuchner, 2011) as well as LB medium (Sambrook and Russel, 2001) supplemented with kanamycin (50 μg ml^−1^) or 5‐FU (20 μg ml^−1^) when required.

### DNA manipulation and strain constructions

The strains, plasmids and primers used in this study are listed in Table 2. The gene targeting vector pKU‐P (Gong *et al*., 2016) used for the integration of *mpd* gene into the chromosome was transformed into *P. putida* KTU by electroporation (Cho *et al*., 1995). Cells were incubated at 30°C for 24 h on LB agar plates containing 50 μg ml^−1^ kanamycin. Positive recombinants occurring with the integration of the plasmid into the chromosome were identified by colony PCR. To promote the excision of the plasmid from the chromosome, the recombinants were incubated in LB medium at 30°C for 12 h. Then, the culture broths that had been diluted to 10^−5^ were spread on LB agar plates supplemented with 20 μg ml^−1^ 5‐FU. Positive recombinants occurring with the exogenous gene replacement were identified by colony PCR. The resulting mutant strain was designated as *P. putida* KTU‐P.

**Table 2:** Strains, plasmids and primers used in this study

Strain, plasmid or primer	Relevant characteristics	Source or reference
*Escherichia coli*		
Trans1 T1	F^‐^, φ80 (*lac*Z), ΔM15, Δ*lac*X74, *hsd*R (r_K_^‐^, m_K_^+^), Δ*rec*A1398, *end*A1, *ton*A	Transgen
*Pseudomonas putida*		
KT2440	Wild type	ATCC 47054
KTU	*upp*‐deficient KT2440	Gong *et al*. (2016)
KTU‐P	KT2440 mutant (Δ*upp*, Δ*pha*A*, mpd* ^*+*^)	This study
KTU‐PG	KT2440 mutant (Δ*upp*, Δ*pha*A, Δ*vdh*,* mpd* ^+^, *gfp* ^+^)	This study
KTU‐PGC	KT2440 mutant (Δ*upp*, Δ*pha*A, Δ*vdh*, Δalg*A*&alg*F*,* mpd* ^+^, *gfp* ^+^, *mcd* ^+^)	This study
Plasmids		
pK18mobsacB	Kan^r^, suicide plasmid for gene knockout	Gong *et al*. (2016)
pKU	Kan^r^, pK18mobsacB containing *upp* gene	This study
pKU‐P	Kan^r^, pK18mobsacB containing *upp* and *mpd*	This study
pKU‐G	Kan^r^, pK18mobsacB containing *upp* and *gfp*	This study
pKU‐C	Kan^r^, pK18mobsacB containing *upp* and *mcd*	This study
Primers		
mpd‐1	5′‐TGGCCTGGAGCTGAAGAACG‐3′	This study
mpd‐2	5′‐CAGTGCAACCACCAGGAGTC‐3′	This study
gfp‐1	5′‐TGGCAGGCGCTGATCTGTTG‐3′	This study
gfp‐2	5′‐TGGCAGATACCCGACTCCAC‐3′	This study
mcd‐1	5′‐AGACTTCCATTGCCAAAGCCCTCAC‐3′	This study
mcd‐2	5′‐ACTGCGCGATGGTCTTCACCGAAAC‐3′	This study
mpd‐F	5′‐GATGCTGCTGGGCGACTTCGAAATC‐3′	This study
mpd‐R	5′‐AAGGCTTGAACTTGCCGGCCTTCAC‐3′	This study
gfp‐F	5′‐ATGGTGAGCAAGGGCGAGGAGCTGT‐3′	This study
gfp‐R	5′‐TTACTTGTACAGCTCGTCCATGCCG‐3′	This study
mcd‐F	5′‐GGGCTCAAGATCTATGTGCCCGAAG‐3′	This study
mcd‐R	5′‐CGCCTTGGTCGATTTGGTCCGATAG‐3′	This study

The construction processes of *P. putida* KTU‐PG and KTU‐PGC were the same as *P. putida* KTU‐P. All the constructed strains were validated by PCR and DNA sequencing. The nucleotide sequences of three synthetic gene cassettes (*mpd*,* gfp* and *mcd*) are shown in Fig. S1. The three synthetic gene cassettes were successively inserted into three different target sites on the chromosome of *P. putida* KT2440. The detailed information on three exogenous genes and their chromosomal insertion sites is shown in Table 1.

### Detection of transcription of exogenous genes by RT‐PCR

Cells of *P. putida* KTU‐PGC were grown overnight in LB medium and harvested. A RNApure Bacteria kit (Cwbio, Beijing, China) was used to acquire total RNA. DNA contamination was eliminated by processing with a DNase I at 37°C for 50 min. RNA integrity was checked by agarose gel electrophoresis. cDNA was prepared using purified total RNA as template and a PrimeScript RT Master Mix kit (Takara, Dalian, China). PCR was carried out with the PrimeSTAR HS DNA polymerase (Takara) using primers listed in Table 2. mRNA and ddH_2_O were used as the templates in control reactions to make sure that DNA products were amplified from cDNA rather than DNA contamination. PCR products were separated by electrophoresis at 80 V on 0.8% Tris Borate EDTA agarose gel stained by ethidium bromide.

### Degradation of carbofuran and CP by *P. putida* KTU‐PGC

Cells of *P. putida* KTU‐PGC were harvested by centrifugation after incubation in 100 ml of LB medium at 30°C for 12 h, washed twice with M9 minimal medium and resuspended (OD_600_ = 1.0) in the same medium. Subsequently, 5 ml of cell suspensions were inoculated into 95 ml of M9 minimal medium supplemented with 100 mg l^−1^ carbofuran and/or 100 mg l^−1^ CP. The samples were incubated at 30°C and 200 rpm in a shaker and withdrawn at regular time intervals. Analysis for the pesticides and their hydrolysis products was performed by HPLC as described below. Degradation experiments were performed with *P. putida* KTU under the same conditions.

### Imaging bacteria

Cells of *P. putida* KTU‐PGC were grown to the plateau stage at 30°C, harvested, washed with PBS buffer (pH 7.2) twice, and then stained with 10 μM FM4‐64/L for 15 min. Cells were fixed with 2% glycerol on a slice, and the observed by a confocal microscope (Leica, Heidelberg, Germany) fitted with a Leica 100 × 10 numerical aperture objective lens, using parameters appropriate for the fluorescence excitation. Expression levels of the GFP were reliably predicted from the fluorescence intensity.

### Soil remediation experiments

Soil samples were collected from the campus of Nankai University, Tianjin, China, which were never exposed to any pesticides before. The soil had a pH of 6.8. Subsamples (100 g) of the soil were treated under aseptic condition with carbofuran (50 mg kg^−1^ soil) and CP (50 mg kg^−1^ soil). Soil samples in triplicate were inoculated with *P. putida* KTU‐PGC (10^6^ cells g^−1^), and soil samples without inoculation were kept as the controls. The inoculum was thoroughly mixed into the soils under sterile condition. The soil moisture was adjusted by the addition of distilled water to 40% of its water‐holding capacity. Soil samples were incubated at 30°C for 12 days in the dark. Soil samples (5 g) were withdrawn for quantifying carbofuran and CP at different time intervals. Carbofuran and CP were extracted from the soil samples using the previous methods described in Jiang *et al*. (2007) and Singh *et al*. (2004). The concentration of carbofuran and CP in the organic extracts was quantified by HPLC as described below.

### HPLC analysis

The culture broths were extracted twice with equal volumes of ethyl acetate. The organic layers containing the pesticides and their hydrolytic products were pooled and then dried over anhydrous Na_2_SO_4_. Aliquots of 20 μl were injected directly to a reversed‐phase HPLC, which was obtained on an Alltech system controller (Alltech Associates, Lexington, Kentucky, USA) equipped with an Innoval ODS‐2 column (Agel, Tianjin, China) and an Alltech UVIS 200 detector. Carbofuran and carbonfuran phenol were detected at 280 nm with methanol/water (50:50, v/v) containing 0.1% phosphoric acid as the mobile phase at a flow rate of 1 ml min^−1^. CP and TCP were analysed at 290 nm with methanol/water (90:10, v/v) as the mobile phase at a flow rate of 1 ml min^−1^. The retention times of the pesticides and their hydrolytic products in HPLC analysis were determined using the authentic standards of each chemical as the reference. The concentration of carbofuran and CP was determined by comparing the peak areas with the calibration curves.

## Supporting information


**Fig. S1.** The nucleotide sequences of the synthetic gene cassettes. (A) *mpd* gene cassette; (B) *gfp* gene cassette; (C) *mcd* gene cassette.
**Fig. S2.** Detection of the introduced exogenous genes in *Pseudomonas putida* KT2440 by agarose gel electrophoresis of PCR products. PCR amplifications were performed with chromosomal DNA as template using primers listed in Table 2. Lane M: DNA marker; lane 1: *mpd* gene cassette; lane 2: *gfp* gene cassette; lane 3: *mcd* gene cassette.
**Fig. S3.** RT‐PCR assays for detecting the transcription of three inserted exogenous genes in *Pseudomonas putida* KT2440. Panels A‐C are the detection results of *mpd*,* gfp* and *mcd* respectively. Lane M: DNA marker; lane 1: control reaction in which genomic DNA was used as template; lane 2: reaction in which cDNA was used as template; lane 3: control reaction in which mRNA was used as template; lane 4: control reaction in which ddH_2_O was used as template.
**Fig. S4.** HPLC analysis of degradation products of carbofuran. *Pseudomonas putida* KTU‐PGC was incubated at 30°C and 200 rpm in a shaker in M9 minimal medium supplemented with 100 mg l^−1^ carbofuran as the sole source of carbon. Carbofuran and carbofuran phenol had a retention time (RT) of 8.75 and 10.21 min respectively. Top, carbofuran degradation detected by HPLC at 0 h; middle, carbofuran degradation detected by HPLC at 6 h; bottom, carbofuran degradation detected by HPLC at 36 h.
**Fig. S5.** Products of degradation of carbofuran and CP by *Pseudomonas putida* KTU‐PGC.
**Fig. S6.** HPLC analysis of degradation products of CP. *Pseudomonas putida* KTU‐PGC was incubated at 30°C and 200 rpm in a shaker in M9 minimal medium supplemented with 100 mg l^−1^ CP as the sole source of carbon. CP and TCP had a retention time (RT) of 8.69 and 3.12 min respectively. Top, CP degradation detected by HPLC at 0 h; middle, CP degradation detected by HPLC at 10 h; bottom, CP degradation detected by HPLC at 24 h.
**Fig. S7.** PCR detection of *mpd*,* gfp* and *mcd* genes in the twentieth‐generation subcultures of *Pseudomonas putida* KTU‐PGC.
**Fig. S8.** Time‐courses for the growth of *Pseudomonas putida* KTU and KTU‐PGC. Cells were incubated in LB medium at 30°C for 48 h. The cell concentration was determined by measuring the OD_600_ of the culture broth.
**Fig. S9.** PCR detection of *mpd*,* gfp* and *mcd* genes in *Pseudomonas putida* SKT‐A (top) and SKT‐B (bottom).
**Fig. S10.** Monitoring of inoculated *Pseudomonas putida* KTU‐PGC by GFP fluorescence using a confocal microscope during soil bioremediation. (A) Green fluorescence within the cell; (B) outline of cell membrane by stain with FM4‐64/L; (C) panels A and B merged together.
