# Supplementary material for: Engineering Pseudomonas putida KT2440 for simultaneous degradation of carbofuran and chlorpyrifos
Source: Microb Biotechnol. 2016 Jul 15;9(6):792–800. doi: 10.1111/1751-7915.12381 (PMC5072195; doi:10.1111/1751-7915.12381)
Supplement: Supplementary file 1 — Fig. S1. The nucleotide sequences of the synthetic gene cassettes. (A) mpd gene cassette; (B) gfp gene cassette; (C) mcd gene cassette. Fig. S2. Detection of the introduced exogenous genes in Pseudomonas putida KT2440 by agarose gel electrophoresis of PCR products. PCR amplifications were performed with chromosomal DNA as template using primers listed in Table 2. Lane M: DNA marker; lane 1: mpd gene cassette; lane 2: gfp gene cassette; lane 3: mcd gene cassette. Fig. S3. RT‐PCR assays for detecting the transcription of three inserted exogenous genes in Pseudomonas putida KT2440. Panels A‐C are the detection results of mpd, gfp and mcd respectively. Lane M: DNA marker; lane 1: control reaction in which genomic DNA was used as template; lane 2: reaction in which cDNA was used as template; lane 3: control reaction in which mRNA was used as template; lane 4: control reaction in which ddH2O was used as template. Fig. S4. HPLC analysis of degradation products of carbofuran. Pseudomonas putida KTU‐PGC was incubated at 30°C and 200 rpm in a shaker in M9 minimal medium supplemented with 100 mg l−1 carbofuran as the sole source of carbon. Carbofuran and carbofuran phenol had a retention time (RT) of 8.75 and 10.21 min respectively. Top, carbofuran degradation detected by HPLC at 0 h; middle, carbofuran degradation detected by HPLC at 6 h; bottom, carbofuran degradation detected by HPLC at 36 h. Fig. S5. Products of degradation of carbofuran and CP by Pseudomonas putida KTU‐PGC. Fig. S6. HPLC analysis of degradation products of CP. Pseudomonas putida KTU‐PGC was incubated at 30°C and 200 rpm in a shaker in M9 minimal medium supplemented with 100 mg l−1 CP as the sole source of carbon. CP and TCP had a retention time (RT) of 8.69 and 3.12 min respectively. Top, CP degradation detected by HPLC at 0 h; middle, CP degradation detected by HPLC at 10 h; bottom, CP degradation detected by HPLC at 24 h. Fig. S7. PCR detection of mpd, gfp and mcd genes in the twentieth‐generation subcu [file MBT2-9-792-s001.doc]

**(A)**

TTTGGATCCCCTGGGGCTGAACCCGGTCATCGGCATCCGCCGCAAGGACCTGTTGAGCTCGGCACGCACCGTGCTGCGCCAGGCCGTGCGCCAACCGCTGCACAGCGCCAAGCATGTGGCTCACTTTGGCCTGGAGCTGAAGAACGTGTTGCTGGGCAAATCCAGCCTGGCCCCGGACAGCGACGACCGTCGCTTCAATGACCCGGCCTGGAGCAACAACCCGCTGTACCGCCGCTACCTGCAAACCTACCTGGCCTGGCGCAAGGAGCTGCAGGACTGGGTGAGCAGCAGCGACCTGTCCCCCCAGGACATCAGCCGCGGCCAGTTCGTCATCAACCTGATGACCGAGGCCATGGCGCCGACCAATACCCTGTCCAACCCGGCTGCGGTCAAACGCTTCTTCGAAACCGGCGGCAAGAGCCTGCTCGATGGCCTGTCCAACCTGGCCAAGGACATGGTCAACAACGGCGGCATGCCCAGCCAGGTGAACATGGATGCCTTCGAAGTGGGCAAGAACCTGGGCACCAGCGAAGGCGCGGTGGTGTACCGCAACGATGTGCTGGAACTGATCCAGTACAGCCCCATCACCGAGCAGGTGCATGCCCGTCCGCTGCTGGTGGTGCCACCGCAGATCAACAAGTTCTACGTGTTCGACCTCAGCCCGGAAAAGAGCCTGGCGCGCTTCTGCCTGCGCTCGCAGCAGCAGACCTTGACAGCTAGCTCAGTCCTAGGTATAATGCTAGCCGCAGTAAGAGAGGAATGTACACATGGCCGCACCGCAGGTGCGCACCTCGGCCCCCGGCTACTACCGGATGCTGCTGGGCGACTTCGAAATCACCGCGCTGTCGGACGGCACGGTGGCGCTGCCGGTCGACAAGCGGCTGAACCAGCCGGCCCCGAAGACGCAGAGCGCGCTGGCCAAGTCCTTCCAGAAAGCGCCGCTCGAAACCTCGGTCACCGGTTACCTCGTCAACACCGGCTCCAAGCTGGTGCTGGTGGACACCGGCGCGGCCGGCCTGTTCGGCCCCACCCTGGGCCGGCTGGCGGCCAACCTCAAGGCCGCAGGCTATCAGCCCGAGCAGGTCGACGAGATCTACATCACCCACATGCACCCCGACCACGTGGGCGGCTTGATGGTGGGTGAGCAACTGGCGTTCCCGAACGCGGTGGTGCGTGCGGACCAGAAAGAAGCCGATTTCTGGCTCAGCCAGACCAACCTCGACAAGGCCCCGGACGACGAGAGCAAAGGCTTCTTCAAAGGCGCCATGGCCTCGCTGAACCCCTATGTGAAGGCCGGCAAGTTCAAGCCTTTCTCGGGGAACACCGACCTGGTGCCCGGCATCAAAGCGCTGGCCAGCCACGGCCACACCCCGGGCCACACCACCTACGTGGTCGAAAGCCAGGGGCAAAAGCTCGCCCTGCTCGGCGACCTGATACTCGTCGCCGCGGTGCAGTTCGACGACCCCAGCGTCACGACCCAGCTCGACAGCGACAGCAAGTCCGTCGCGGTGGAGCGCAAGAAGGCCTTCGCGGATGCCGCCAAGGGCGGCTACCTGATCGCGGCGTCCCACCTGTCGTTCCCCGGCATCGGCCACATCCGCGCCGAAGGCAAGGGCTACCGTTTCGTGCCGGTGAACTACTCGGTCGTCAACCCCAAGTGAGCTGCTGACTCTGTAAGAGAGGAATGTACACATGACCGTCACCGATATCATCCTGATCCACGGCGCCTTGAACCGCGGCGCCTGCTATGACGCGGTCGTCCCGCTTCTCGAAGCGCGCGGCTACCGCGTCCATGCGCCCGACCTGACCGGCCATACGCCCGGCGATGGCGGCCATTTGTCGGTCGTCGACATGGAGCATTATACCCGCCCAGTCGCTGACATCCTGGCACGGGCCGAGGGGCAGTCGATCCTTCTGGGGCACAGCTTGGGCGGTGCATCCATCTCGTGGCTGGCGCAGCACCATCCCGACAAGGTGGCCGGGCTGATCTACCTGACCGCGGTCCTCACCGCGCCCGGTATAACGCCGGAAACCTTCGTCCTGCCCGGCGAGCCCAACCGGGGCACGCCGCACGCGCTGGACCTGATCCAGCCGGTCGACGAGGGACGTGGGCTACAGGCGGATTTCTCGCGACTGGAACGGCTCCGCGAAGTTTTCATGGGCGATTATCCCGGCGAGGGAATGCCGCCTGCCGAACAGTTCATCCAGACCCAGTCGACCGTGCCCTTTGGCACGCCCAATCCGATGGAGGGGCGCGCGCTGGAAATCCCACGCCTCTATATCGAGGCGCTGGACGATGTCGTGATTCCGATCGCCGTGCAGCGTCAGATGCAGAAGGAGTTCCCCGGTCCGGTCGCGGTCGTGTCGCTGCCGGCCAGCCATGCGCCCTATTACTCGATGCCCGAACGGCTGGCCGAGGCGATCGCCGATTTCGCCGATGCCCCGGCCGAGTATCGCCAGACGGCGACGAAGGCTGGGCCTGATCGACCAGCTGGAGCGGACGGGGGTCGAGCCGACCGAGCTGATCTACCGTGAGCCATTGGCGAGAACAAGGTCAACGCCCTGACCCTGCTGGTCAGCGTGCTGGACACCACCATGGACAACCAGGTTGCTTTGTTTGTCGACGAGCAGACCTTGGAGGCCGCCAAGCGCCACTCCTATCAGGCGGGCGTGCTGGAAGGCAGCGAAATGGCCAAGGTGTTCGCCTGGATGCGCCCCAACGACCTGATCTGGAACTACTGGGTAAACAACTACCTGCTCGGCAATGAGCCCCCCGTGTTCGACATCCTGTTCTGGAACAACGACACCACGCGCCTGCCGGCCGCCTTCCACGGCGACCTGATCGAAATGTTCAAGAGCAACCCGCTGACCCGCCCCGACGCCCTGGAAGTGTGCGGCACCGCGATCGACCTGAAACAGGTCAAATGCGACATCTACAGCCTCGCCGGCACCAACGACCACATCACCCCCTGGCCGTCATGCTACCGCTCGGCACATCTGTTCGGCGGCAAGATCGAATTCGTACTGTCCAACAGCGGGCATATCCAGAGCATCCTCAACCCGCCGGGCAACCCGAAGGCACGTTTCATGACCGGTGCCGATCGCCCGGGTGACCCGGTGGCCTGGCAGGAAAATGCCATCAAGCATGCAGACTCCTGGTGGTTGCACTGGCAGAGTTGGCTGGGCGAGCGTGCCGGCGCGCTGAAAAAGGCACCGACCCGCCTGGGCAACCGTAAAGCTTTTT

**(B)**

TTTGGATCCATGTTGCAGGTGCCTTTGCTGATTGGCGGGCAGTCGCGCCCCGCCAGCGATGGACGAACCTTCGAGCGCTGTAACCCGGTGACTGGCGAGGTGGTGTCGCAGGCTGCCGCCGCCACACTGGCCGATGCCGATGCCGCGGTGGCTGCTGCCAGCGCGGCGTTTCCGGCCTGGGCCGCCCTGGCACCGGGCGAGCGGCGCAGCCGCTTGCTGGCAGGCGCTGATCTGTTGCAGGCGAGGGCCGCCGAGTTCATCGCCGCCGCCGGTGAAACCGGGGCCATGGCCAACTGGTATGGCTTCAACGTGAAGTTGGCCGCCAACATGCTGCGCGAGGCTGCAGCCATGACCACGCAGATCACCGGTGAAGTGATCCCCTCGGACGTTCCCGGCAGCTTCGCAATGGCCCTGCGCGCGCCCTGCGGCGTGGTGTTGGGCATCGCACCGTGGAACGCCCCGGTGATACTGGCCACGCGTGCCATTGCCATGCCGCTGGCCTGCGGCAACACCGTGGTGCTCAAGGCCTCGGAGCTGAGCCCGGCGGTCCATCGGCTGATCGGCCAGGTGCTGCACGATGCAGGCATCGGCGACGGCGTGGTCAATGTCATCAGCAATGCGCCGCAGGATGCCCCCGCCATCGTCGAGCGGCTGATCGCCAACCCTGCGGTACGCCGGGTCAACTTCACCGGTTCGACGCACGTCGGGCTTGACAGCTAGCTCAGTCCTAGGTATAATGCTAGCCGCAGTAAGAGAGGAATGTACACATGGAAACGCTAGATGGAGTCGTTGTAGTTGGCGGTGGTCCGGTTGGACTTCTTACTGCACTGAAACTGGGTAAAGCCGGTATTAAAGTCGTTGTGCTCGAAGCGGAGCCAGGCGTCTCGCCTTCGCCGCGCGCGGTCGCCTACATGCCACCGACCGCCGCCGCGCTGGATCGCTTCGGCCTGCTCCAAGACATCCGCAAGCGTGCGGTGATGTGCCCGGATTTCGCCTATCGCCATGGCAACGGTGAGCTGATCGCGAAGATGGACTGGAGCGTCCTGTCCCAGGACACCCAGTATCCGTACATGCTGTTGCTGGGGCAGAACCACGTGTCCAACGTGATTTTCCAGCACCTGCGAGAGCTGCCGAACGTCGAGATCCGCTGGAATCACCGGGTCGAGGAAGTCGACCAGGACGACGCCTACGTGACCATTGAGACCAGTAGCCCCGGGGGCACATCACGCCTGCGTGCACGTTGGCTGGCGGCCACCGACGGTGCCCGCAGCACCGTGCGGCAGAAGATCGGCCTGACCTTCGACGGGATTACCTGGGATGAGCGTCTGGTCGCCACCAACGTCTTTTATGACTTCTCGCTGCATGGCTACTCGCGCGCCAACTTCGTCCACGATCCGGTGGACTGGGCCGTGGTCGTGCAACTGGACAAGACCGGCCTGTGGCGCGTCTGTTATGGCGAAGACGCTTCGTTGTCCGACGCCGAAGTTCGCCGTCGCCTGCCCGAGCGCTTCAAGCGTTTGTTGCCGGGGGCGCCGACGCCGGACCAGTACCGCGTCGATCACCTCAACCCGTATCGCGTTCACCAGCGCTGCGCGGCGGAGTTCCGGCGTGGGCGAGTGGTGCTGGCGGGCGATGCGGCCCACGCCACCAACCCGATGGGCGGCCTTGGTCTGTCCGGCGGGGTGCTGGATGCCGAACACCTGGCTGAGGCCCTGATCGCCGTGATCAAGAACGGTGCATCGACCAAGACCCTGGATGAATACTCGATCGACCGTCGCAAGGTCTTCCTCGAATTCACCTCGCCAACCGCCACCGCCAACTTTACCTGGATGAAGGAAAGCGATCCGGCGCAACGCATCCGTGACGATGCGATGTTCAAGGAAGCGGGGACCGATCGGGCAGTCATGCGCCAGTTCCTGCTGGACCTCGAAAAACTCAATGGCCGCCGTGTCATAGAAAAAAAGCTTAAAGCCGCCTGAGCTGCTGACTCTGTAAGAGAGGAATGTACACATGCCAACAAAAATTCAGATCGTTTTCTACAGCTCCTATGGCCACATCTACAAAATGGCCGAAGCCATCGCTGCCGGCGCGCGCGAAGTCGGTGATGTCGAAGTCACCCTGTTGCAAGTGCCGGAACTGATGCCTGAAGAGGTTCAGGTCAAGAGCGGGATCAAGGGCTACCGCGCTGCGTTCGGCAGCATCCCTTACGCAACGCCTGAGGTTTTGGCTGAAGCGGATGCCATCATTTTTGGTACTCCGACCCGCTTCGGCAACATGTGCAGCCAAATGCGCAACTTCCTCGACCAGACTGGCGGCCTGTGGATGTCCGGCGGCTTGATCGGCAAAGTCGGCAGCGTGTTCACCAGCACTGCTTCGCAGCACGGCGGCCAGGAAACCACCATCACCAGCTTCCACACCACGCTGCTGCACCACGGTATGGTCATCGTTGGCGTTCCTTACTCTGAACCAGGCCTGACCAACATGACCGAGATCTCGGGCGGTACGCCTTACGGCGCCTCCACCCTGGCCGGTGCCGACGGTTCGCGTCAGCCAAGCGAAAACGAGCTGCAGATCGCGCGCTTCCAGGGCAAGCACGTGGCGACCATCGCCAAGCGCCTGGCGAACAACAAGTAAGCTGCTGACTCTTTGACAGCTAGCTCAGTCCTAGGTATAATGCTAGCCGCAGTAAGAGAGGAATGTACACATGGTGAGCAAGGGCGAGGAGCTGTTCACCGGGGTGGTGCCCATCCTGGTCGAGCTGGACGGCGACGTAAACGGCCACAAGTTCAGCGTGTCCGGCGAGGGCGAGGGCGATGCCACCTACGGCAAGCTGACCCTGAAGTTCATCTGCACCACCGGCAAGCTGCCCGTGCCCTGGCCCACCCTCGTGACCACCCTGACCTACGGCGTGCAGTGCTTCAGCCGCTACCCCGACCACATGAAGCAGCACGACTTCTTCAAGTCCGCCATGCCCGAAGGCTACGTCCAGGAGCGCACCATCTTCTTCAAGGACGACGGCAACTACAAGACCCGCGCCGAGGTGAAGTTCGAGGGCGACACCCTGGTGAACCGCATCGAGCTGAAGGGCATCGACTTCAAGGAGGACGGCAACATCCTGGGGCACAAGCTGGAGTACAACTACAACAGCCACAACGTCTATATCATGGCCGACAAGCAGAAGAACGGCATCAAGGTGAACTTCAAGATCCGCCACAACATCGAGGACGGCAGCGTGCAGCTCGCCGACCACTACCAGCAGAACACCCCCATCGGCGACGGCCCCGTGCTGCTGCCCGACAACCACTACCTGAGCACCCAGTCCGCCCTGAGCAAAGACCCCAACGAGAAGCGCGATCACATGGTCCTGCTGGAGTTCGTGACCGCCGCCGGGATCACTCTCGGCATGGACGAGCTGTACAAGTAAACTGGGCGGCAAGGCACCTTTGCTGGTGCTCGACGATGCCGACCTGGACGCCACGGTCGAAGCGGCGGCCTTCGGTGCCTACTTCAACCAGGGGCAGATCTGCATGTCCACCGAGCGCCTTGTGGTGGACAGCTGTATTGCCGACGCTTTCGTCGACAAGCTGGCGGTGAAGATCGCCGGGCTGCGTGCAGGTGATCCGCAAGCCAGCACCTCGGTGCTCGGCTCGCTGGTCAGCGCAGCGGCCGGCGAGCGCATCAAGGCACTGATCGACGATGCCGTGGCCAAGGGCGCGCGCCTGGTCAGCGGCGGCCAGCTGGAAGGCAGCATCCTGCAACCGACCTTGCTCGACAACGTCGATGCCAGCATGCGCCTGTACCGCGAGGAGTCCTTCGGCCCGGTGGCGGTGGTACTGCGCGCCGAAGGCGACGAAGCCTTGCTGCAGCTGGCCAACGACTCGGAGTTCGGTCTGTCATCGGCCATTTTCAGCCGCGACACCAGCCGCGCCCTGGCCTTGGCCCAACGGGTGGAGTCGGGTATCTGCCATATCAACGGCCCGACCGTTCACGATGAAGCGCAGATGCCGTTTGGCGGGGTCAAGTCCAGCGGCTATGGCAGCTTCGGCAGCCGCACGGCCATCGATCAGTTCACCCAGTTGCGCTGGGTCACCCTCCAGCACGGCCCGCGTCACTATCCCATCTAGGGATCCTTT

**(C)**

TTTGAATTCATGACTACCAAGACTTCCATTGCCAAAGCCCTCACCCTCGCGGCCGGCCTTTCCCTTGCTTCGATGCAGGCCTTCGCTGGTGCCGACGCCGCACTGTATGGCCCAAGCGCGCCGAAAGGCTCGACCTTCGTACGCCTGTACAACGCGACCAGCGCACCGGCCGCCGCGTCGGTCGGCAACACCCAGATCAAACAGGTGGGCGCACAGGCCAGCAGCGACTTCAGCTTCCTGCCAGGCGGCGACTACACCGCCCAGGTCGGCGGCAAGAGCGTGCCGGTCAAGCTGGCCTCGGACAAGTACTACACCCTGGTCAACAGCAACAGCGGCAGCCCGAAACTGATCGAAGAACCACCGTTCAAGAACAAGCAGAAAGCCCTGGTGCGCGTGCAGAACCTGAGCGACCAGCAACTGACCCTGAAAACCGCCGACGGCAAGACCGAAGTGGTCAAGCCGGTGGCCGCCAACGGCCGTGGCGAACGCGAAATCAACCCGGTCAAGGTCAACCTGGCGCTGTACCAAGGAGACAAGAAAGTGGGTGACGTGAAACCCGTCGCCCTGGAGCGCGGCGAAGCCGCAGTGCTGTACGTAACGGGTTCCGGCTTGACAGCTAGCTCAGTCCTAGGTATAATGCTAGCCGCAGTAAGAGAGGAATGTACACATGGCCTTGAATCGTATCAAGAACTCAAATCCATCTACCTTGCCCCCTGAAACTCCCGGCGAAGGGCCCTTCATGCACGAGCATCAAGCAGACATTCTCAAATGGATCAGGCTCGCTGACGACGTCTACCGCTTTCGCGACAGCTGCAACGTCTACGCACTGATCGGCGACTCGGGCAGCGTCATCATCGACGCCGGTACCGGGGCGTGGCTCGATCATGTCGAGCAATTGCCGAAAGAGCCCGCCGCGCTTCTGTGCACGCACTTCTTCAGAGATCACGCCGAGGGCGCGGCCTACGCCGCCCGGGCCGGGCTCAAGATCTATGTGCCCGAAGCCGAGTTGGACATATTTGTCGATCCCCATATGCATCACCTTCAGAGGGAGACGATTTGCAGCTTTGATTCGGTCTATTGGCATCATTTCGCGCCGATCGAACCCGTCGCGATCGCCGGTGTCCTCAAGGATCACGAAATCCTAGAGCTCGCTGGCTTGAAGCTCGAGGTGGTTCCCCTTCCGGGCGCGACAATCGGTCAGATCGGAGTCGCCTTCCTATCGTCCGAACTTGGGTCCGTCCTGTGCAGTGCCGAGACGATTCATTCCGAGGGAAGGATCCCGCGGATTGCGCCCCTCCAGCAGGTCTATGTCGATCTTGACGGGATCGCCATGGTCTATGGCAGCGTCCGTGCCCTTTCGCTCAGAAAGGTCGACGTGCTGCTTCCGAGCCTAGGCGAACCCATTCTTGCCGGGGTCGATCGCTGCCTGTCCATGCTTTCGGCCAACCTGCGCAAGGCCGCCGGCCGACGCATGAACGGGGGAGAGACGGTTCCGCTCGCCTTCCTGCTCGACGCGATCGACGACACCGCTCTGGTCCGGATCAGCGAGCACGTCTATCGGACCAAATCGACCAAGGCGACGACTGCGTTCCTTGTCAGCCAATCCGGCAAGGTTCTCAGCATTGACTATGGCGCGCCTCACCACATGCTGAACTACTCGATCCTCGGCCGACGGTCCACGCGGCGAGGGATGCTGCACAGCCTCGATGAGTTGGAGGCGATCACGGGGCGCCGTGGGATAGATGTCGTGCTAACGACCCATTTTCACGACGACCATGTCGGCGGCATCAATCTTCTGAAGAGGGTGTTCGGAACAAAGGTCTGGGCGTCGAAGGCCTACTCGGAAATTCTTGAAAAACCCGGCGACCGACTGCTCCCGGCGGCCTGGCCCTTCCCGATCGCCGTCGACCGTGTACTGGAACATGATGAAGTCTTCCGCTGGGAGGAGTTCGAGTTCCGAATGGGACCGGAAATCGGGGGCGGCCACACCCACCACCAGGCGGTCTGTTCGTTCGTTGCTGATGGACTGACCTACGCGGCAGTCGGGGACCAATATCTCAGCCGCAAGCTCTGGAACCCCCAACCGGACCACGATTGGCAAGACGATGAATGGGACGATGTCTTTTGCTATCGCAGCGGCCAAACCGCAACGGGCTACAGGCTCAGCGAGAACTGGCTTATCGACCTTCGCCCGGACGTCATATTGAACGGGCACCAGCCCGCGATCATGACGAACGACCTCGTCTTTCAGCGGGTGACGGAAATGTCGGCGCGATTTGAGAACCTGCATCGAAGCCTCATGCCACTCGACGAGAATGACGAACATTTCGGCCTCGACTCGACAGGCGCGTGGATCGAACCCTACAGGCTTCACAGGCTGGAACCGGGGCAGACGTGCGTTCGTATTCGGGTGCGAAACCCCCTACCGCGCGCGGCCACGCTTGAGCTTCGCATGTCCGGGGCCGCGGTCCTCGTGGACAAGGCCGTTGCTGTCACTTGTGTGGGCCATGAAGAGGCGGTTTTCGAGGCGACCGTTCTGCTCCCCCGCACTTGCAGGCGAGAACCGGCTGCTCTTTCGATGTGGGCGCAGGGAAGGCCCTTCGGTCAGGTGTCGGAGGTCCTGGTCACCGTCGGTCACGCCCGATGGTGAAACGGCAACGTCACCAAGGGCGACGTGGTGGTTCAAGACAGCCGCAACTGCATGATCCACGGCAACGGCAAACTGGTGTCGGTGATCGGCCTGGAGAACATCGTGGTGGTCGAGACCAAGGATGCCATGATGATTGCCCACAAGGACAAGGTCCAGGGCGTCAAGCAGATGGTCAAGACCCTCGACGAACAGGGCCGCACGGAAACCCAGAACCACCTGGAAGTGTATCGCCCGTGGGGCTCGTACGACTCGGTGGACATGGGCGGCCGCTTCCAGGTCAAGCACATCACCGTCAAGCCGGGCGCCAGCCTCTCGCTGCAGATGCACCACCACCGCGCCGAACACTGGATCGTGGTATCCGGTACCGCCGAGGTGACCTGCGACGAGAACGTGTTCTTGCTGACCGAAAACCAGTCGACCTACATCCCCATCGCTTCGGTGCACCGTTTGCGCAACCCGGGCAAGATCCCGCTGGAGATCATCGAGGTGCAGTCCGGGAGCTACCTGGGCGAGGATGACATCGAGCGCTTCGAGGATGTGTATGGGCGCACGTCCACCCCCATCGAGCGCGGCGTTTCGGTGAAGACCATCGCGCAGTAAGAATTCTTT

**Fig. S1.** The nucleotide sequences of the synthetic gene cassettes. **(A)** *mpd* gene cassette; **(B)** *gfp* gene cassette; **(C)** *mcd* gene cassette.


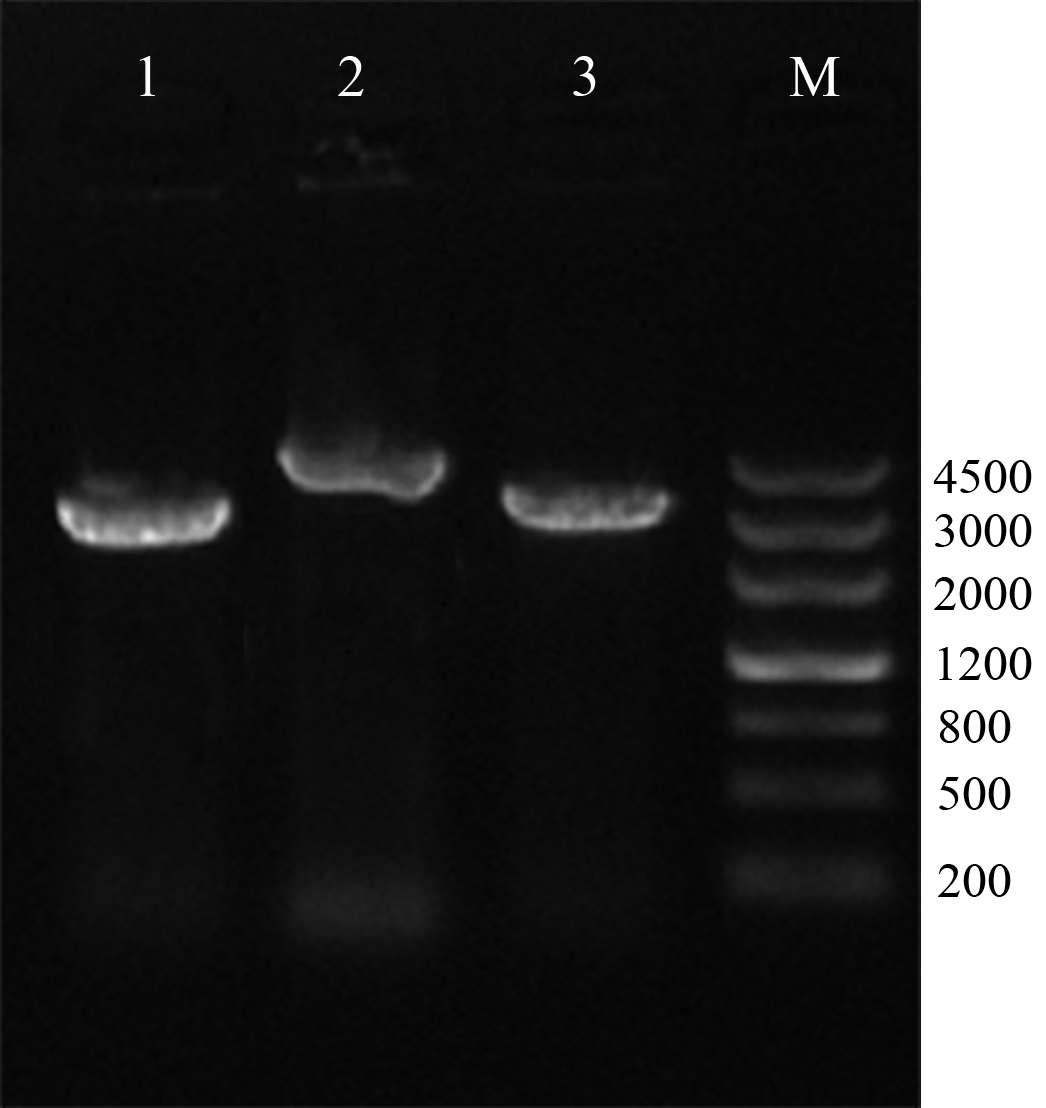


**Fig. S2.** Detection of the introduced exogenous genes in *P. putida* KT2440 by agarose gel electrophoresis of PCR products. PCR amplifications were performed with chromosomal DNA as template using primers listed in Table 2. Lane M: DNA marker; lane 1: *mpd* gene cassette; lane 2: *gfp* gene cassette; lane 3: *mcd* gene cassette.


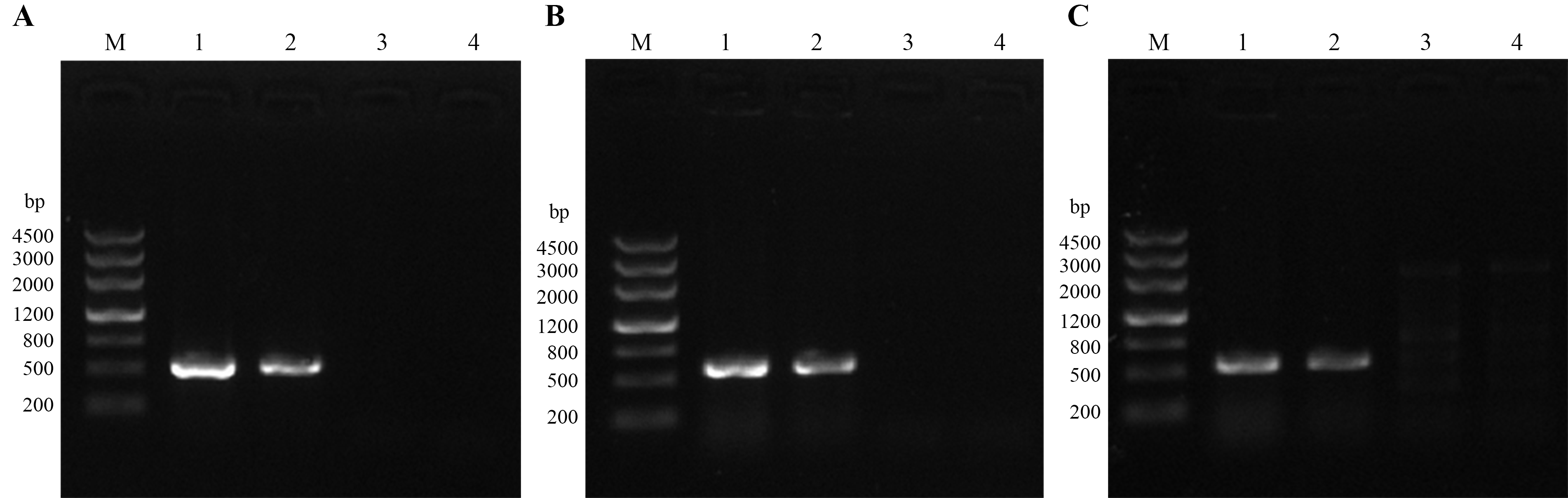


**Fig. S3.** RT-PCR assays for detecting the transcription of three inserted exogenous genes in *P. putida* KT2440. Panels A−C are the detection results of *mpd*, *gfp* and *mcd*, respectively. Lane M: DNA marker; lane 1: control reaction in which genomic DNA was used as template; lane 2: reaction in which cDNA was used as template; lane 3: control reaction in which mRNA was used as template; lane 4: control reaction in which ddH_2_O was used as template.


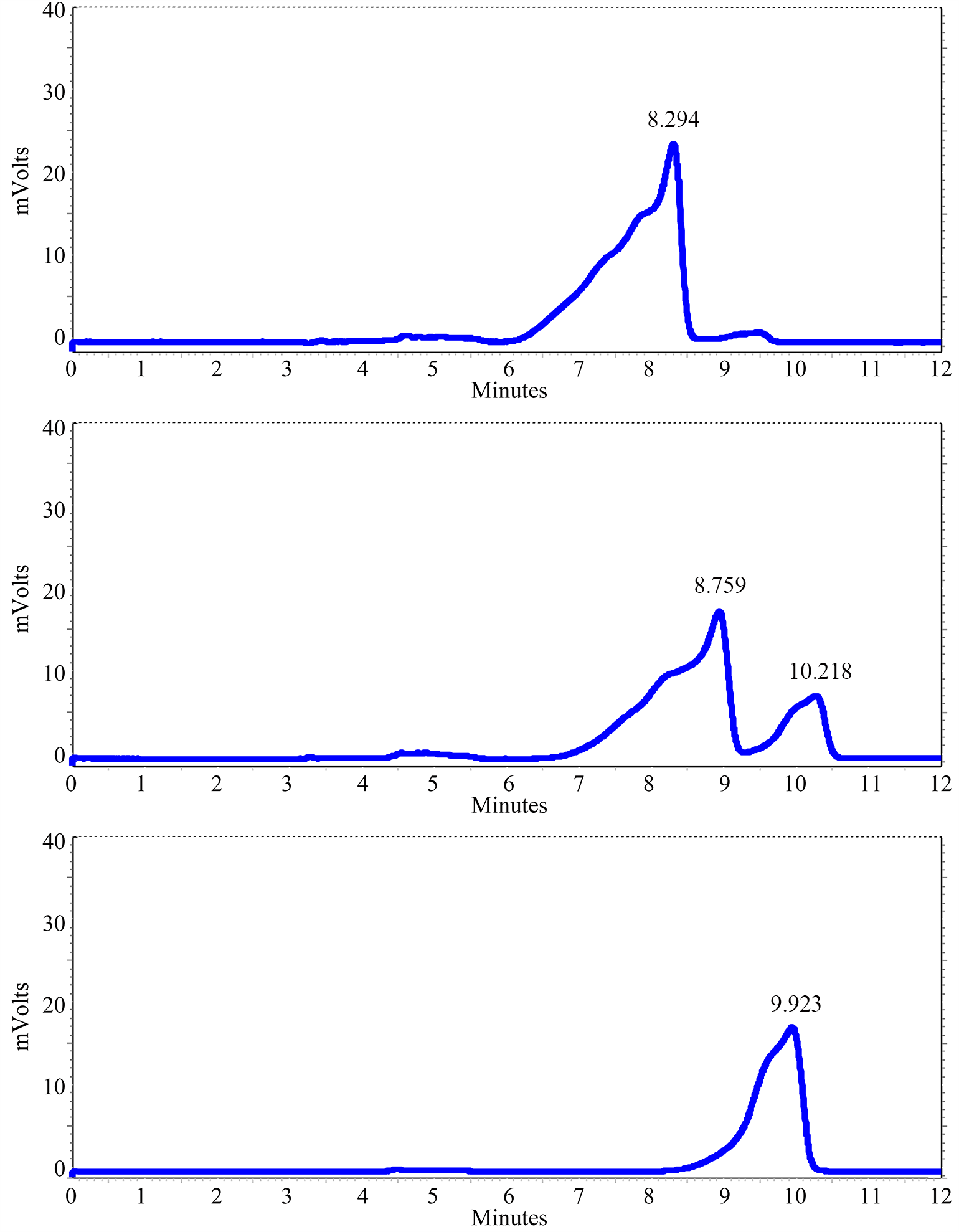


**Fig. S4.** HPLC analysis of degradation products of carbofuran. *P. putida* KTU-PGC was incubated at 30°C and 200 rpm in a shaker in M9 minimal medium supplemented with 100 mg/l carbofuran as the sole source of carbon. Carbofuran and carbofuran phenol had a retention time (RT) of 8.75 and 10.21 min, respectively. Top, carbofuran degradation detected by HPLC at 0 h; middle, carbofuran degradation detected by HPLC at 6 h; bottom, carbofuran degradation detected by HPLC at 36 h.


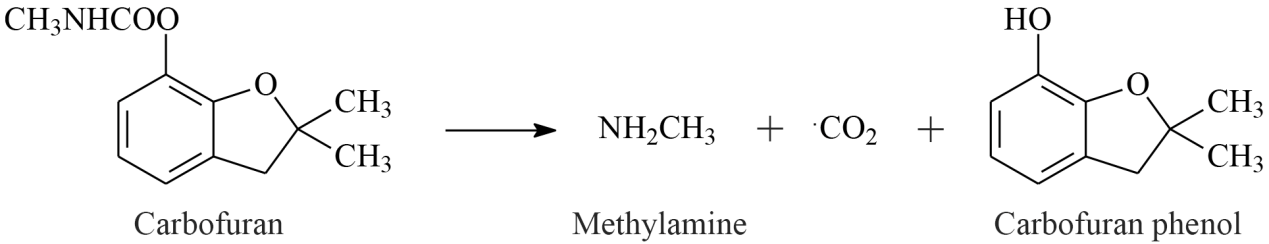


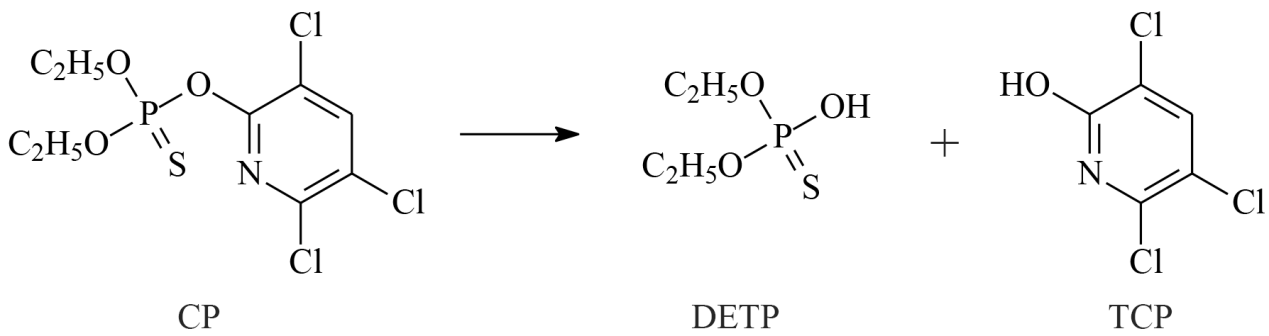


**Fig. S5.** Products of degradation of carbofuran and CP by *P. putida* KTU-PGC.

**
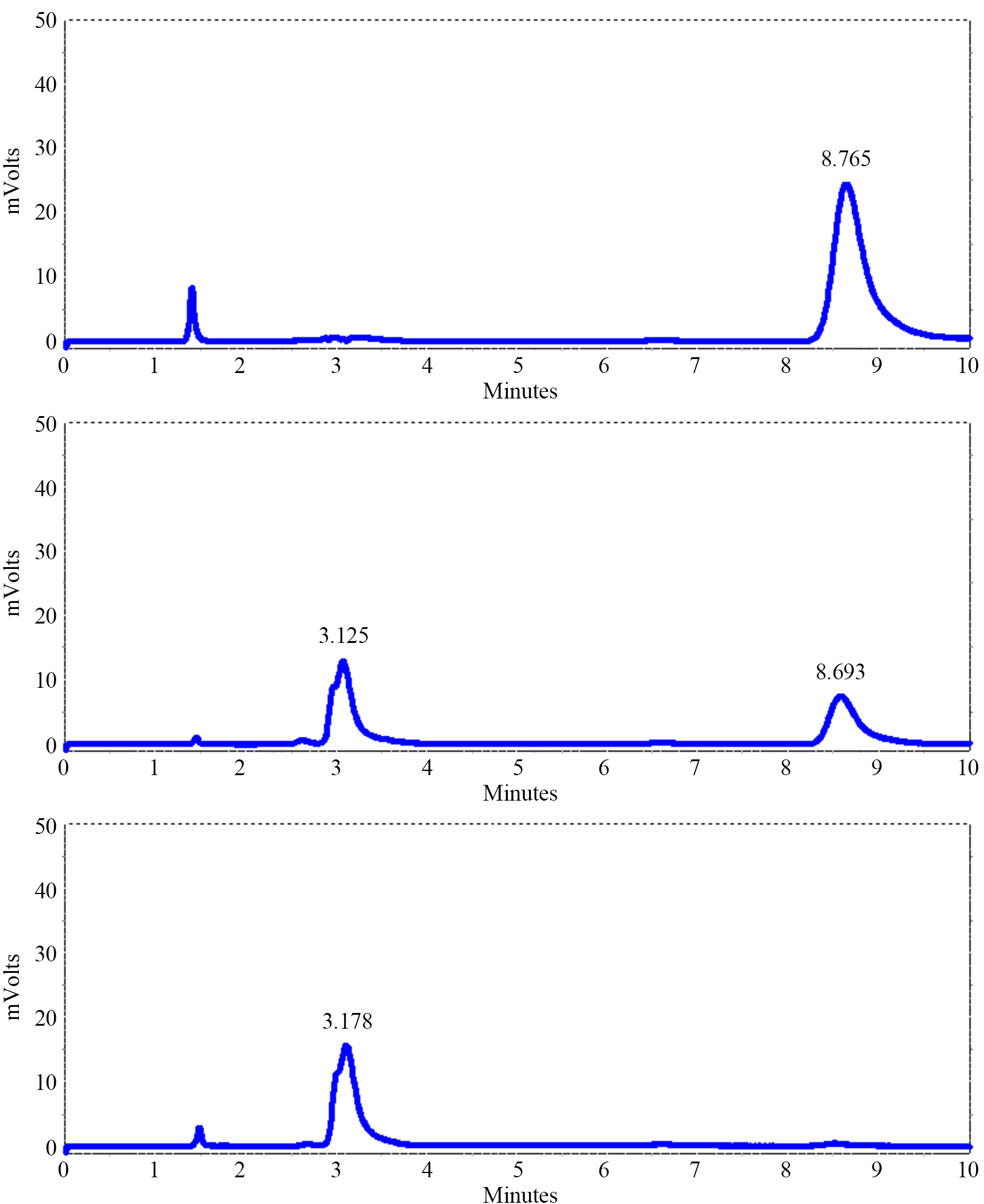
**

**Fig. S6.** HPLC analysis of degradation products of CP. *P. putida* KTU-PGC was incubated at 30°C and 200 rpm in a shaker in M9 minimal medium supplemented with 100 mg/l CP as the sole source of carbon. CP and TCP had a retention time (RT) of 8.69 and 3.12 min, respectively. Top, CP degradation detected by HPLC at 0 h; middle, CP degradation detected by HPLC at 10 h; bottom, CP degradation detected by HPLC at 24 h.


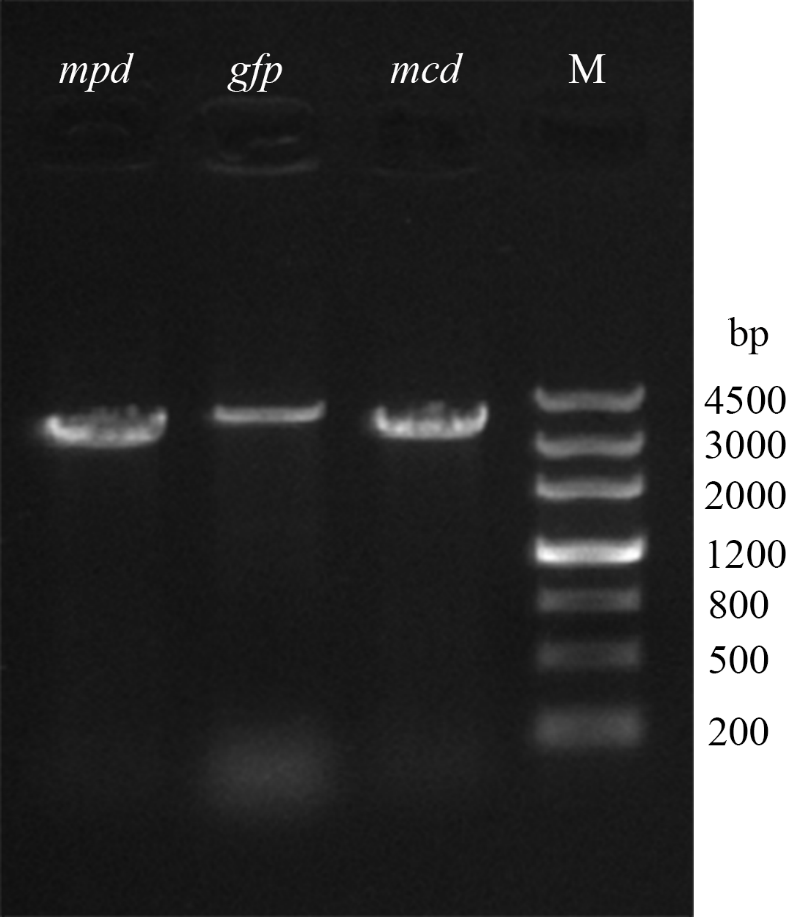


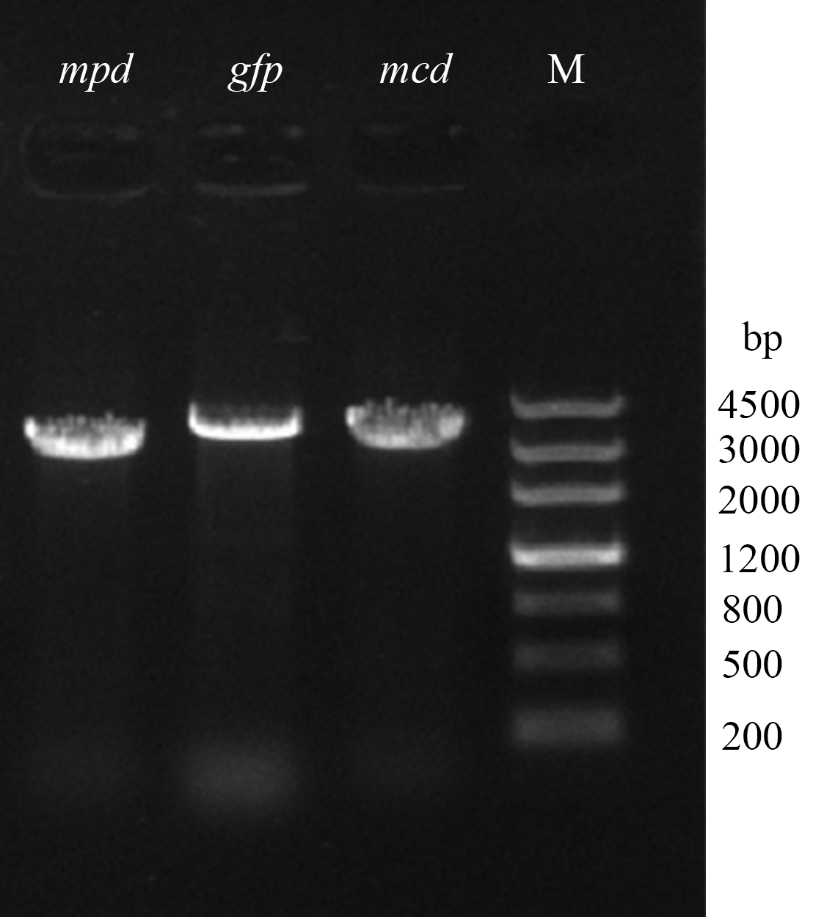


**Fig. S7.** PCR detection of *mpd*, *gfp* and *mcd* genes in the twentieth-generation subcultures of *P. putida* KTU-PGC.


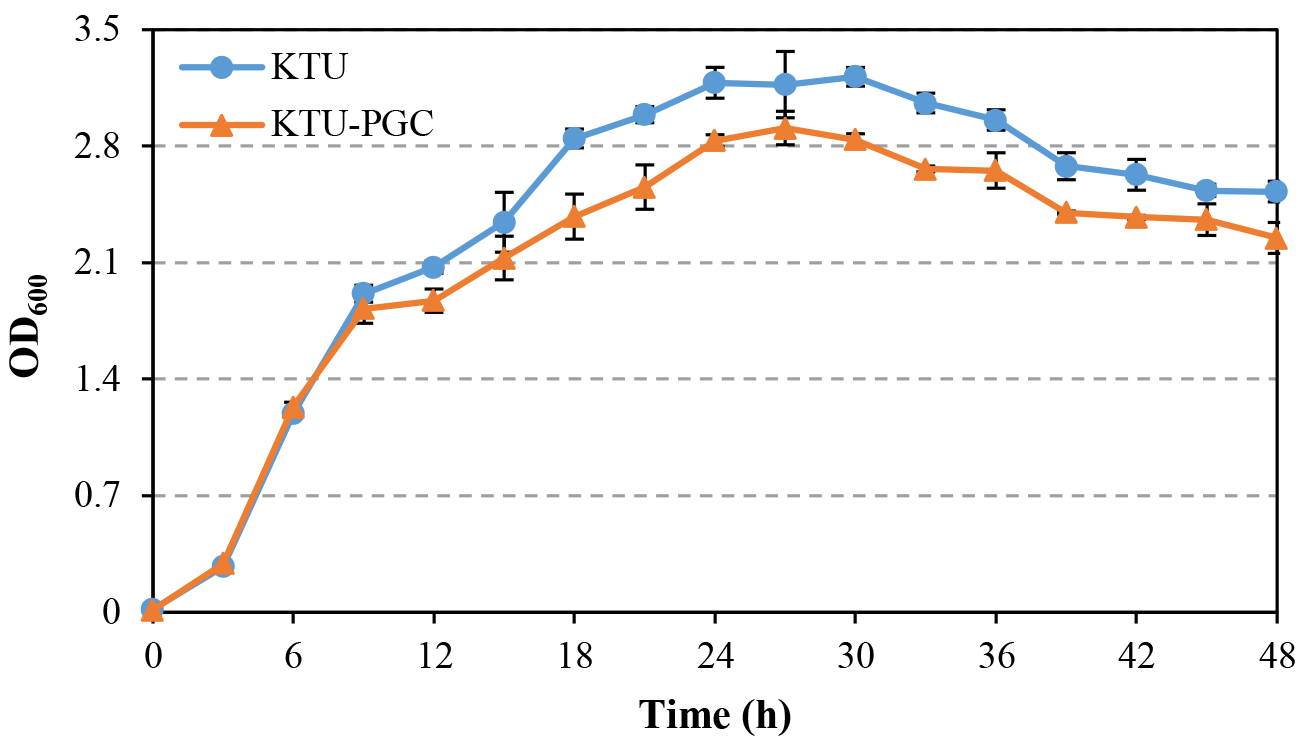


**Fig. S8.** Time courses for the growth of *P. putida* KTU and KTU-PGC. Cells were incubated in LB medium at 30°C for 48 h. The cell concentration was determined by measuring the OD_600_ of the culture broth.


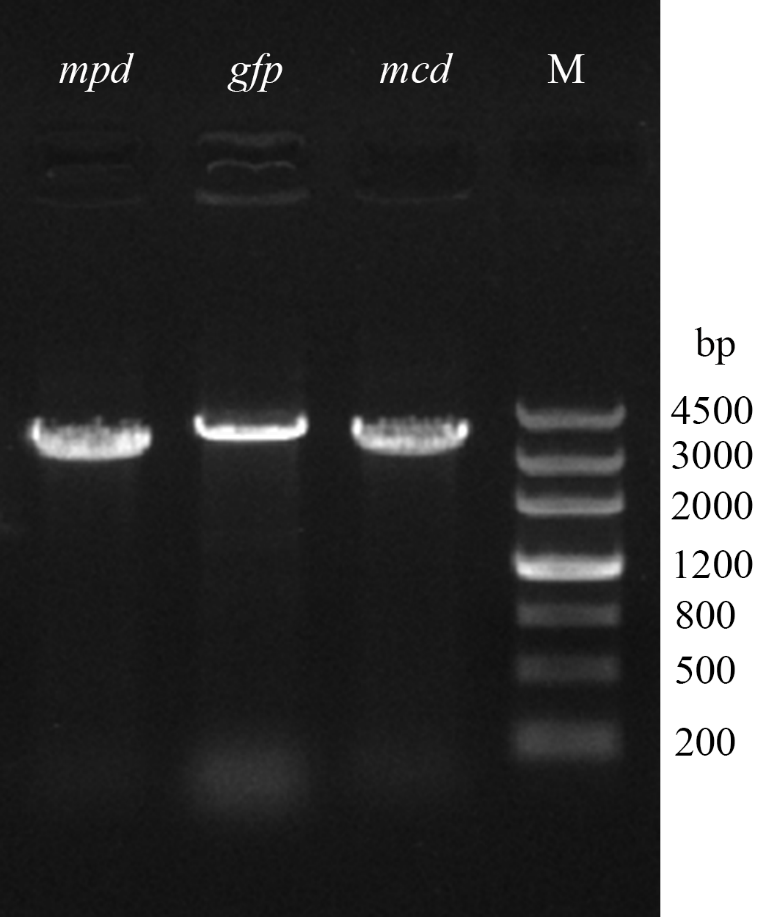


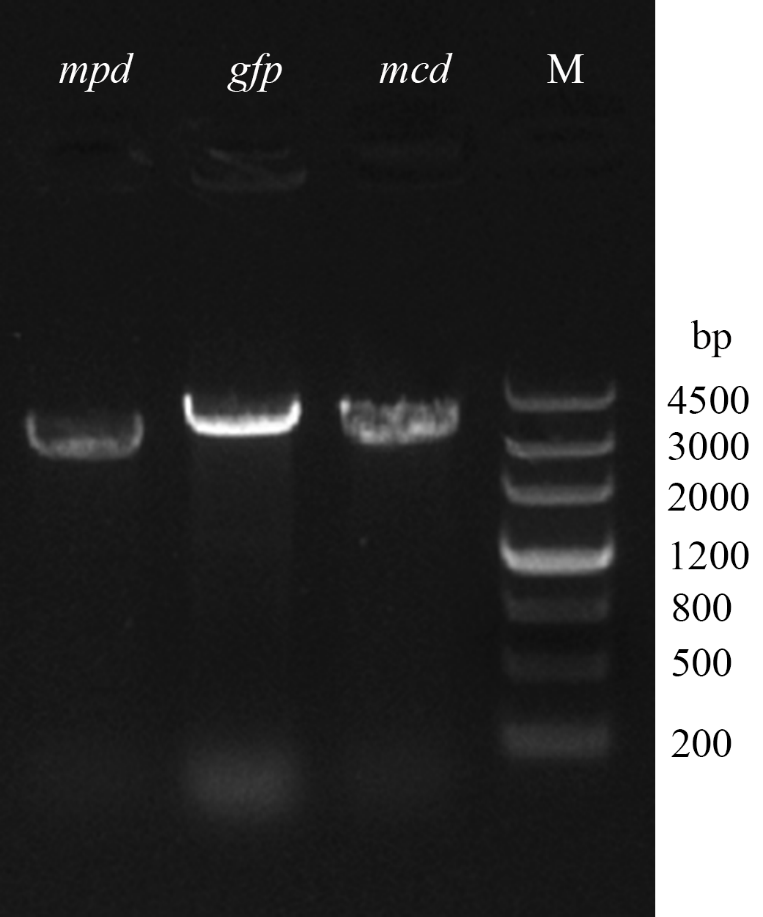


**Fig. S9.** PCR detection of *mpd*, *gfp* and *mcd* genes in *P. putida* SKT-A (top) and SKT-B (bottom).


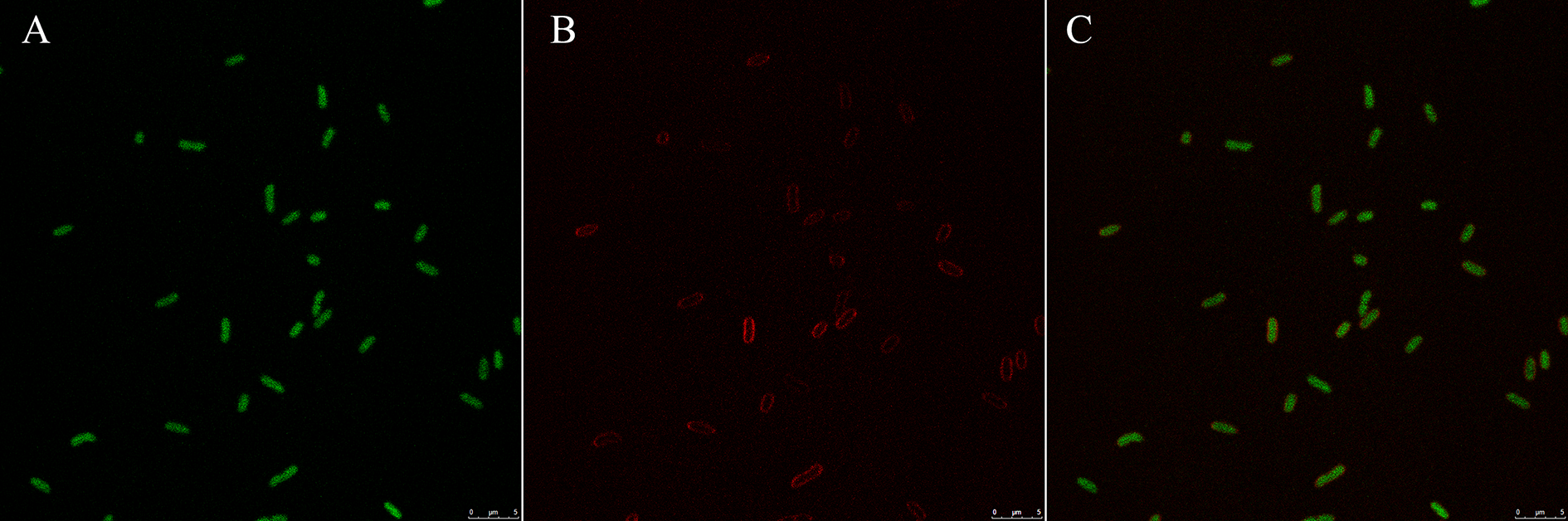


**Fig. S10.** Monitoring of inoculated *P. putida* KTU-PGC by GFP fluorescence using a confocal microscope during soil bioremediation. (A) Green fluorescence within the cell; (B) outline of cell membrane by stain with FM4-64/L; (C) panels A and B merged together.
